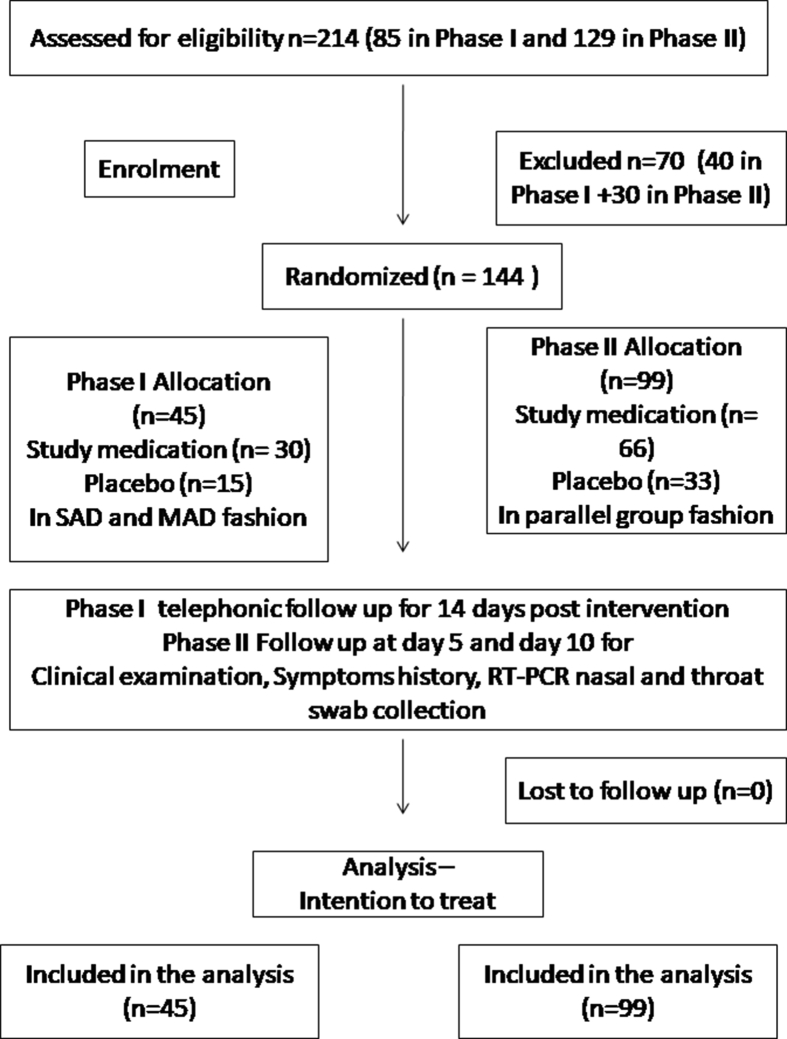# Essential oil nebulization in mild COVID-19(EONCO): Early phase exploratory clinical trial

**DOI:** 10.1016/j.jaim.2022.100626

**Published:** 2022-07-06

**Authors:** Ramya Rathod, Ritin Mohindra, Akshay Vijayakumar, Roop Kishor Soni, Ramandeep Kaur, Ankit Kumar, Naveen Hegde, Aishwarya Anand, Swati Sharma, Vikas Suri, Kapil Goyal, Arnab Ghosh, Ashish Bhalla, Nanda Gamad, Amrit Pal Singh, Amol N. Patil

**Affiliations:** a Department of Otolaryngology(Head & Neck Surgery), PGIMER, Chandigarh, India; b Department of Internal Medicine, PGIMER, Chandigarh, India; c Department of Virology, PGIMER, Chandigarh, India; d Department of Pharmacology, PGIMER, Chandigarh, India; e Department of Experimental Medicine and Biotechnology, PGIMER, Chandigarh, India; f Department of Dravyaguna, Shri Dhanwantry Ayurvedic College, Chandigarh, India

**Keywords:** Eucalyptus oil, Peppermint oil, Tea tree oil, COVID-19, Symptomatic relief, Safety, Efficacy

## Abstract

**Background:**

Medications studied for therapeutic benefits in coronavirus disease 2019 (COVID-19) have produced inconclusive efficacy results except for steroids.

**Objective:**

A prospective randomized open-label, parallel-arm Phase I/II clinical trial was planned to compare essential oil (EO) blend versus placebo nebulization in mild COVID-19.

**Methods:**

A Phase I safety evaluation was carried out in a single ascending and multiple ascending dose study designs. We assessed Phase II therapeutic efficacy on COVID-19 and general respiratory symptoms on days 0, 3, 5, 7, 10, and 14 on the predesigned case record form. Viremia was evaluated on day 0, day 5, and day 10.

**Results:**

Dose-limiting toxicities were not reached with the doses, frequencies, and duration studied, thus confirming the formulation's preliminary safety. General respiratory symptoms (p < 0.001), anosmia (p < 0.05), and dysgeusia (p < 0.001) benefited significantly with the use of EO blend nebulization compared to placebo. Symptomatic COVID-19 participants with mild disease did not show treatment benefits in terms of symptomatic relief (p = 1.0) and viremia clearance (p = 0.74) compared to the placebo. EO blend was found to be associated with the reduced evolution of symptoms in previously asymptomatic reverse transcription polymerase chain reaction (RT-PCR)-positive study participants (p = 0.034).

**Conclusion:**

EO nebulization appears to be a safer add-on symptomatic relief approach for mild COVID-19. However, the direct antiviral action of the EO blend needs to be assessed with different concentrations of combinations of individual phytochemicals in the EO blend.

## 1 Introduction

The world is currently facing multiple waves of coronavirus disease 2019 (COVID-19) pandemic, caused by severe acute respiratory syndrome coronavirus 2 (SARS-CoV-2). Most of the developed and developing countries have introduced vaccine strategy. However, still many countries are struggling with the production of the vaccines, their outreach issues, and social acceptability leading to the persistence of the pandemic. The efficacy evidence on the use of synthetic antivirals, antibiotics, and other antimicrobials to control SARS-CoV-2 is limited at present [1]. In this regard, the utilization of essential oils (EOs) to control the global pandemic situation can be assessed against COVID-19, just like its proven efficacy against other infectious diseases [2].

EOs constitute a significant source of innovative therapeutic agents isolated from different plant species that contain compounds such as lectins, polypeptides, alkaloids, phenols, and quinine. These have shown potent antimicrobial activity against a broad spectrum of microorganisms. EOs and their components may interact with protein synthesis, enzymes, or cofactors and inhibit viral DNA or RNA synthesis. Many plant-based EOs, extracts, and individual phytochemical ingredients have been demonstrated to possess antiviral efficiency against enveloped and non-enveloped viruses [3]. There appear to be multiple mechanisms for antiviral action of plant-derived antimicrobials. Nevertheless, the majority of these antimicrobials appear to act either directly on the virus itself (e.g., on the envelope or capsid) or during the early stages of virus replication following internalization of the virus into its host cell [4].

EOs and their active phytochemicals were extensively studied against severe acute respiratory syndrome coronavirus 1 (SARS-CoV-1), SARS-CoV-2, Middle East respiratory syndrome coronavirus (MERS-CoV), rhinovirus, herpes simplex virus 1 and 2 (HSV-1 and HSV-2), human immunodeficiency virus (HIV), coxsackievirus (CV), enterovirus (EV71), hepatitis B virus (HBV), hepatitis C virus (HCV), influenza virus (H1N1) and respiratory syncytial virus (RSV) [5]. Evidence from several in vitro studies has formed the basis for the potential antiviral efficacy of EOs. In addition, anti-inflammatory, mucolytic, bronchodilator, and immunomodulatory activities are also mentioned in the Ayurveda literature. Therefore, EOs could be useful as a prophylactic, adjunctive, alternative, or as an integrated approach to decrease the morbidities associated with COVID-19 and enhancing host immunity against SARS-CoV-2 [6,7]. Thus far, the antiviral activity of these phytopharmaceuticals is studied only with the help of in vitro studies. The literature lacks its translational value assessment in patients affected with infectious diseases. The purpose of the current study was to explore early-phase safety and efficacy evidence of the EO blend in mild COVID-19 patients.

## 2 Methods

### 2.1 Study design

The present study was an open-label, sequential (for Phase I), parallel-arm (for Phase II), randomized, placebo-controlled, and clinical trial. The percentage and weight by volume composition of the EO blend are presented in the supplementary file Table 1. The EO blend drops mixed in five mL normal saline (NS) nebulization was the intervention arm, while plain five mL NS nebulization alone was kept as the placebo arm.

### 2.2 Ethical considerations

The study was performed in August 2021 after obtaining due approval from the ethics committee via sanction communication [INT/IEC/2021/SPL/1012 dated 26.06.2021]. The trial was registered with the clinical trial registry of India (CTRI) with identifier no. CTRI/2021/07/034,962. Written informed consent was obtained from all study participants before enrolment in the study—the study abided by the ethical principles laid down by the Declaration of Helsinki.

### 2.3 Plant material

The EO plant species, namely lemon oil, oregano oil, tea tree oil, java citronella oil, turmeric oil, peppermint oil, lavender, ginger, frankincense, eucalyptus, wheat germ, basil, cedarwood, holy basil, cinnamon oil, sage oil, and clove oil, were identified and confirmed by phytochemist Dr. ALV Kumar. Plants were obtained from different parts of India. The EO blend (registered batch no. 250320211) was prepared in a good manufacturing practice (GMP)-certified laboratory. The study formulation used here is a certified formulation for various clinical indications in Ayurveda practice by the Department of AYUSH (Ayurveda, Yoga & Naturopathy, Unani, Siddha, and Homeopathy), which recognizes the traditional system of medicine in India, vide approval product ID No. L-318/Ayur/0046/2021/P via letter No. 11595/DA/2021 dated 16 June 2021.

For future reference, a voucher specimen (voucher no. RR/PGI/Pharma/EO001/21) was submitted to room no. 4028 of the Department of Pharmacology, PGIMER.

#### 2.3.1 Preparation of plant material, standardization, and chromatographic determination

The standard steam distillation method, cold press method, and oil soak method were chosen for plant EO extraction. The reverse-phase HPLC-PDA strategy was chosen to check the ingredients in the final mixture (Supplementary file Fig. 1). YMC PROPAK C-18 (150 × 4.6 um x 1.8 u) column and mobile phase A consisting of 0.1% formic acid in the water and mobile phase B made up of acetonitrile:methanol (50:50) was finalized from the literature. Separation was achieved through gradient elution from −20% B to 90% B in 35 min, followed by 90% B back to 20% in 40 min. The flow rate was kept at 0.7 mL/min. The column temperature was set at 25 °C with detection wavelengths studied at 220 nm (the most common wavelength covering the maximum number of phytochemicals), 254 nm, 265 nm, 275 nm, and 435 nm for encompassing all phytochemical constituents (Supplementary file Fig. 1).

The study was performed in two stages: Phase I and Phase II time cohorts.

### 2.4 Common randomization, blinding, and allocation concealment plan for all subsets of the study population

A 2:1 randomization design (two study participants received EO blend as against one received placebo) was opted for in the single ascending dose (SAD), multiple ascending dose (MAD) designs, and Phase II stage of the study. The requisite number of randomization codes were prepared in Microsoft Excel 2007 using the “rand” function.

Sealed pack envelopes contained two codes for the study medication (i.e., EO blend in five mL NS nebulization) and placebo (i.e., five mL NS nebulization alone). Each pack was plan to be opened just before the nebulization session. The study medication and placebo vials were of the same dark amber-colored opaque medium, helping in the physician and endpoint assessor's blinding.

The study participants could not be blinded due to the aroma of EOs, but the response assessor was blinded by distance of one meter between patient and physician in well ventilated room. The statistician was blinded throughout the study.

### 2.5 Phase I clinical trial

#### 2.5.1 Inclusion and exclusion criteria

**Fig. 1:**
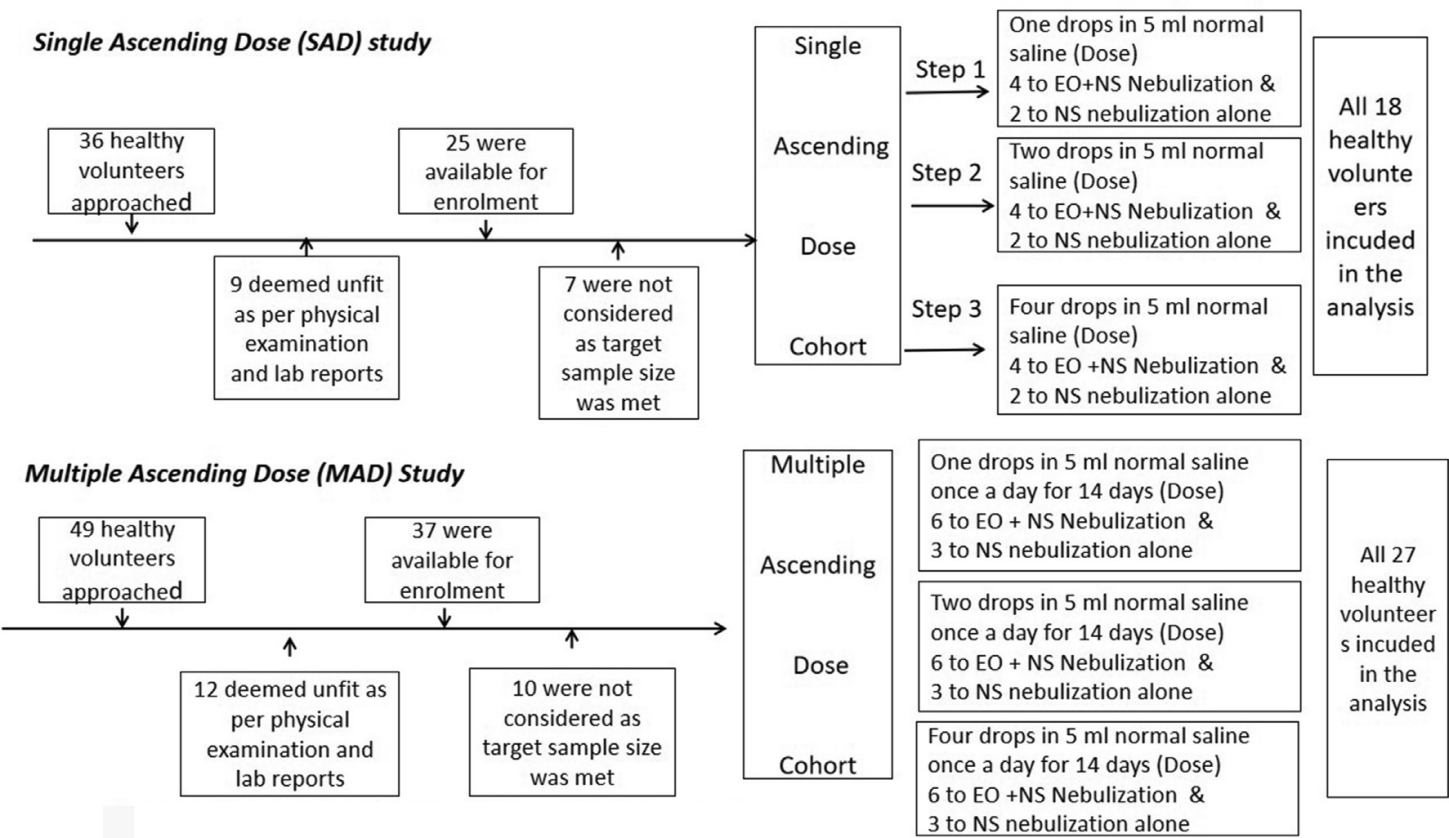
Phase 1 trial design inclusive of single ascending dose (SAD) and multiple ascending dose (MAD) approach.

Healthy volunteers of either gender, aged between 18 and 55 years, were the inclusion criteria for Phase I of the clinical trial (Fig. 1). However, any health concerns (potentially confounding the endpoint assessment) confirmed by physical examination by the investigator physician was set as the predefined exclusion criterion.

Inclusion and exclusion criteria remained the same for the SAD and MAD sequential sections of the Phase I study.

#### 2.5.2 Single ascending dose (SAD) study

We planned healthy volunteers in the experimental arm to take the study medication (EO blend, single dose) with the adult dose range, as observed from the naturalistic Ayurveda practice for other ailments (i.e., one, two, and four drops in five mL NS via nebulization).

One healthy volunteer received one dose, either study medication or placebo, at the given time. It was scheduled to increase to a higher dose level (in another set of healthy volunteers) once the safety evaluation of the prior set cohort of six volunteers was complete and satisfactory (Fig. 1).

#### 2.5.3 Multiple ascending dose (MAD) study

Healthy volunteers received study medication intervention (i.e., one, two, and four drops in five mL NS via nebulization of EO blend) once a day for 14 consecutive days.

For 14 consecutive days, the same dose strengths (one drop, two drops, and four drops of EO blend in 5 mL NS) were administered. The participants were followed up telephonically till one week after drug administration was completed (Fig. 1).

#### 2.5.4 Safety endpoint assessment

The study planned to evaluate safety concerns, if any, for three dose strengths and causality was assessed using the Naranjo scale and the Hartwig–Siegel scale. Study participants were observed 10 min before, 10 min during, and 2 h post nebulization.

The study had a predefined the stopping rule (i.e., if the study participant develops a hypersensitivity reaction, rash, breathlessness, chest discomfort, hypotension or blood pressure falling below 100/70, declining oxygen saturation below 93%, respiratory rate rising >30 per minute, and heart rate increasing to more than 110/min). Any permutation or combination of symptoms mentioned here was searched.

### 2.6 Phase II study

The Phase II study design was an open-label, parallel arm, and randomized controlled clinical trial.

#### 2.6.1 Sample size calculation

In the absence of a previous similar study, the probability was assumed to be 80 percent to detect a treatment difference at a one-sided significance level of 5%, with the true difference between treatments kept 0.5 times the standard deviation. The calculated sample size derived was 90. Assuming an attrition rate of 10%, 100 participants were required in the Phase II study. No formal sample size calculation was done for the Phase I study.

#### 2.6.2 Inclusion and exclusion criteria

Phase II study design inclusion criteria were patients of either gender, aged between 18 and 65 years, consenting for participation, diagnosed SARS-CoV-2 infection with RT-PCR test, asymptomatic or mild disease symptomatic patients with oxygen saturation ≥93% at the time of presentation to the emergency room, and who had oxygen saturation not deteriorating upon a 6-min walk test. Exclusion criteria were set as any significant medical or surgical illness that may interfere with the study endpoint assessment as per the discretion of the investigator team and pregnant and lactating females.

#### 2.6.3 Intervention for phase II study

A safe dose of two drops of EO blend in five mL NS for ten days was considered against a same volume of placebo for the Phase II part of the study.

The study arms were labeled as follows:

**AT arm:** Asymptomatic patients receiving treatment medication.

**AP arm**: Asymptomatic patient receiving placebo.

**ST arm:** Symptomatic patients receiving treatment medication.

**SP arm:** Symptomatic patient receiving placebo.

The patient was given ample opportunity to understand and ask questions related to the ultrasonic nebulization procedure which was followed once a day for 14 days.

#### 2.6.4 Efficacy endpoint assessment

Patients were educated to remain adherent to the treatment protocols and timely lab investigations, in addition to the telephonic and physical follow-up assessment for symptom progression or remission.

##### 2.6.4.1 Symptomatic remission or progression assessment

Phase II study participants (arm AT, AP, ST, and SP) were evaluated for symptomatic relief on days 0, 3, 5, 7, and 10. Symptom assessments included fever, cough, breathlessness, chest discomfort, fatigue, lethargy, myalgia/arthralgia, nausea, vomiting, diarrhea, anosmia, dysgeusia, headache, syncope, and altered sensorium, which were noted at follow-up time points. Anosmia and dysgeusia were assessed on a visual analog scale, while the Borg scale was used for breathlessness assessment if present. Other symptoms were noted as categorical variables absent or present at any time of the follow-up day.

##### 2.6.4.2 Virological assessment

Patients called for viremia assessment by RT-PCR testing on days 0, 5, and 10. We collected nasal and oropharyngeal swab samples from the study participants, and an RT-PCR test was carried out against the E and ORF genes. The results of the RT-PCR test were planned to be obtained in both a qualitative (positive, negative, inconclusive) and quantitative (cycle threshold [CT] score) manner. The higher the CT score, the milder the disease. CT more than 36 is assumed as “negative.” We collected data on a predesigned case record form for each participant.

The Consolidated Standards of Reporting Trials (CONSORT) reporting checklist was followed during the conduct and compilation of the entire trial data (Supplementary file Fig. 2).

**Fig. 2:**
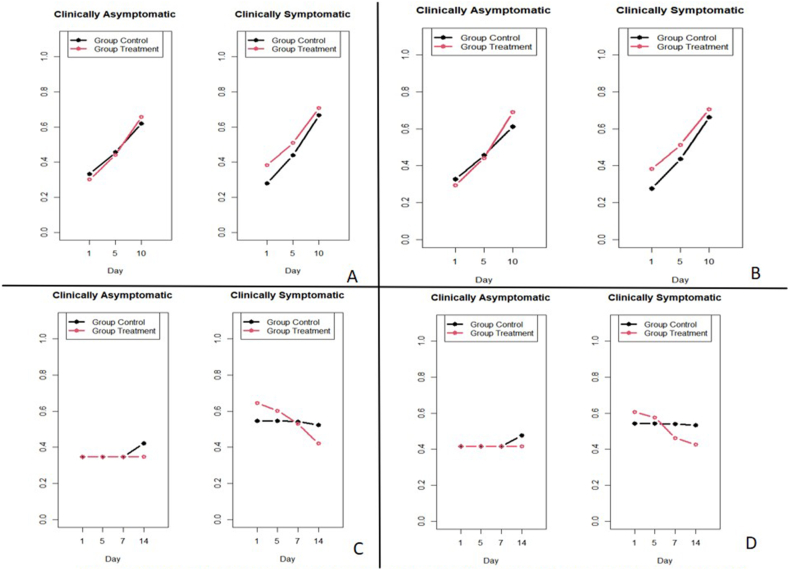
Plots of relative treatment effect (RTE) on E gene (A), ORF gene(B), Anosmia (C), dysguesia (D).

#### 2.6.5 Statistical analysis

IBM SPSS Version 26.0 and Microsoft Office Excel 2007 were used. Continuous data were expressed as median ± interquartile range. Categorical data were analyzed using the chi-square test or Fisher's Exact test. Repeated measure logistic regression using generalized estimating equations was used to analyze the categorical variables with repeated measures over time. A p-value < 0.05 was accepted as statistically significant. An intention-to-treat analysis was planned for the Phase II subsection of the study.

## 3 Results

A total of 144 participants (45 healthy volunteers for phase I and 99 COVID-19 positive patients for phase II) entered the present study.

### 3.1 Phase I study findings

Forty-five healthy volunteers participated in the Phase I section of the study. Eighteen healthy volunteers in the SAD study and twenty-seven in the MAD study sections saw good tolerability with the administration's dose, frequency, and duration. One patient complained of a mild burning sensation on day 3 of the six-step cohort (Fig. 1). The adverse event subsided in 5 min without requiring any rescue treatment or further investigation. Dose-limiting toxicity was not achieved in the present study.

### 3.2 Phase II study findings

It included 99 RT-PCR-positive COVID-19 patients. In four study arms, all participants’ E gene and ORF gene CT scores were equal to or less than 36 at baseline. Sixteen patients were enrolled in Arm AT, eight in Arm AP, 50 in Arm ST, and 25 in Arm SP.

Baseline attributes and past history details showed nonsignificant differences between EO nebulization (study medication) versus placebo subgroups (Table 1). Vaccination status before study enrolment is presented in supplementary file Table 2. The mild COVID-19 disease symptoms showed nonsignificant difference in symptom distribution at baseline between the two groups, as presented in Table 3.

**Table 1:** Baseline characteristics of the study participants in each arm [Frequency (Percentage)].

Basic characteristics	Arm AT	Arm AP	Arm ST	Arm SP	Total	p value
Gender						
Male	7 (43.8)	5 (62.5)	25 (50)	13 (52)	50 (50.5)	0.874
Female	9 (56.3)	3 (37.5)	25 (50)	12 (48)	49 (49.5)	
Age	35.5 (25–47)	41 (37–43.5)	38 (29–45)	38 (31–58)	38 (29–45.5)	0.695
Weight (in kg)	55 (47–63.5)	70 (56.5–77.5)	65 (58–72)	70 (62–78)	65 (57–74.5)	0.06
Height (in cm)	159 (143.3–165)	167.5 (165–177)	165 (158–170)	165 (163–170)	165 (158–170)	0.23
BMI	23.4 (19.3–25.8)	24.1 (22–27.9)	24.1 (21.8–26.4)	26 (23.9–26.5)	24.2 (22.2–26.5)	0.166
**Family History of COVID-19**						
Yes	10 (62.5)	2 (25)	25 (50)	8 (32)	45 (45.5)	0.149
No	6 (37.5)	6 (75)	25 (50)	17 (68)	54 (54.5)	
**Travel History**						
Yes	2 (12.5)	2 (25)	6 (12.2)	1 (4)	11 (11.2)	0.339
No	14 (87.5)	6 (75)	43 (87.8)	24 (96)	87 (88.8)	
**Co-morbidities**						
**Diabetes**						
Present	2 (12.5)	0 (0)	6 (12)	5 (20)	13 (13.1)	0.612
Absent	14 (87.5)	8 (100)	44 (88)	20 (80)	86 (86.9)	
**Hypertension**						
Present	2 (12.5)	0 (0)	3 (6)	4 (16)	9 (9.1)	0.413
Absent	14 (87.5)	8 (100)	47 (94)	21 (84)	90 (90.9)	
**Coronary artery disease**						
Present	0 (0)	0 (0)	1 (2)	1 (4)	2 (2)	1.000
Absent	16 (100)	8 (100)	49 (98)	24 (96)	97 (98)	
**Chronic Kidney disease**						
Present	1 (6.3)	0 (0)	0 (0)	0 (0)	1 (1)	0.242
Absent	15 (93.8)	8 (100)	50 (100)	25 (100)	98 (99)	
**Liver disease**						
Present	1 (6.3)	0 (0)	2 (4)	0 (0)	3 (3)	0.677
Absent	15 (93.8)	8 (100)	48 (96)	25 (100)	96 (97)	
**Thyroid disorder**						
Hypothyroidism	3 (18.8)	1 (12.5)	4 (8)	0 (0)	8 (8.1)	0.098
None	13 (81.3)	7 (87.5)	46 (92)	25 (100)	91 (91.9)	
**HIV**						
Yes	0 (0)	0 (0)	1 (2)	0 (0)	1 (1)	1.000
No	16 (100)	8 (100)	49 (98)	25 (100)	98 (99)	
**Addiction**						
Yes	1 (6.3)	0 (0)	4 (8)	1 (4)	6 (6.1)	0.916
No	15 (93.8)	8 (100)	46 (92)	24 (96)	93 (93.9)	
**Psychiatric disorder**						
Yes	0 (0)	1 (12.5)	2 (4)	0 (0)	3 (3)	0.268
No	16 (100)	7 (87.5)	48 (96)	25 (100)	96 (97)	

p < 0.05 assumed significant.

**Table 2:** Development of new symptoms in the asymptomatic study participants over a follow up period and assessment of remission of respiratory symptoms in asymptomatic as well as symptomatic participants.

Treatment arms	Time/Follow-up					p value
	Development of new symptoms in the study participants					
	Day 3	Day 5	Day 7	Day 10	Day 14	
AT (n = 16)	2 (12.5)	0 (0)	0 (0)	0 (0)	0 (0)	0.034∗
AP (n = 8)	3 (37.5)	0 (0)	0 (0)	0 (0)	2 (25)	
	**Day 3**	**Day 5**	**Day 7**	**Day 10**	**Day 14**	
ST (n = 50)	0 (0)	0 (0)	1 (2)	1 (2)	0 (0)	1.00
SP (n = 25)	0 (0)	0 (0)	0 (0)	0 (0)	1 (4)	
Remission of the respiratory symptoms in the study participants						

Treatment arms	Day 3	Day 5	Day 7	Day 10	Day 14	
ST (n = 50)	37 (74)	47 (94)	47 (94)	49 (98)	50 (100)	0.000000001
SP (n = 25)	1 (4)	3 (12)	5 (20)	12 (48)	21 (84)	

P < 0.05 assumed significant.

**Legends:** AT -Asymptomatic patient receiving treatment drug; AP- Asymptomatic patient receiving placebo; ST - Symptomatic patient receiving treatment drug; SP-symptomatic patient receiving placebo.

**Table 3:** Symptoms of COVID-19 in the study participants in symptomatic arms C (ST) and D (SP).

Symptoms	Arm C (ST)	Arm D (SP)	Total	p value
**Breathlessness**				
Present	1 (2)	1 (4)	2 (2.7)	1.000^b^
Absent	49 (98)	24 (96)	73 (97.3)	
**Fatigue**				
Present	20 (40)	15 (60)	35 (46.7)	0.102^a^
Absent	30 (60)	10 (40)	40 (53.3)	
**Myalgia**				
Present	8 (16)	5 (20)	13 (17.3)	0.750^b^
Absent	42 (84)	20 (80)	62 (82.7)	
**Nausea/Vomiting/Diarrhoea**				
Present	2 (4)	2 (8)	4 (5.3)	0.597^b^
Absent	48 (96)	23 (92)	71 (94.7)	
**Anosmia**				
Present	26 (52)	9 (36)	35 (46.7)	0.190^a^
Absent	24 (48)	16 (64)	40 (53.3)	
**Dysgeusia**				
Present	18 (36)	6 (24)	24 (32)	0.294^a^
Absent	32 (64)	19 (76)	51 (68)	
**Other Symptoms**^c^				
Chest Discomfort	2 (4)	2 (8)	4 (5.3)	0.597^b^
Flu like symptoms	6 (12)	2 (8)	8 (10.7)	0.711^b^
Sore throat	13 (26)	2 (8)	15 (20)	0.066^a^
Headache	3 (6)	0 (0)	3 (4)	0.546^b^
Depression	1 (2)	0 (0)	1 (1.3)	1.000^b^
Nasal blockage	1 (2)	0 (0)	1 (1.3)	1.000^b^
None	29 (58)	19 (76)	48 (64)	–

ST - Symptomatic patient receiving treatment drug; SP-symptomatic patient receiving placebo.

∗Significant at 0.05 level.

a Chi-square test used.

b Fisher's Exact test used.

c Multiple symptoms seen in some participants. Fever episode was present in almost all symptomatic participants in both the subgroups.

Asymptomatic individuals receiving study medication and placebo were assessed for symptom progression on days 3, 5, 7, and 10 of follow-up. The evolution of new symptoms assessed during the follow-up period included breathlessness, fatigue, myalgia, nausea, vomiting, diarrhea, anosmia, dysgeusia, chest discomfort, headache, soreness in the throat, nasal block, fever, and cough. The repeated-measure logistic regression using generalized estimating equations showed a significant difference (p = 0.034) between the two treatment groups in the evolution of new symptoms (Table 2). Similarly, symptomatic COVID-19 positive participants were followed up for specific COVID-19 symptom remission differences. A statistically nonsignificant difference was observed between the two subgroups (p = 1.0).

Furthermore, EO nebulization was assessed for general respiratory symptom relief in the symptomatic mild disease participants from arms ST and SP. It showed a statistically significant difference for respiratory symptom relief (p < 0.000000001). The effects on viremia and core COVID-19 symptoms were also assessed for the two interventions. A statistically insignificant difference was observed in viremia concerning CT scores calculated for the E (p = 0.74) and ORF (p = 0.579) genes. However, asymptomatic (arms A and B) and symptomatic (arms C and D) subgroups showed statistically significant effects on anosmia (p = 0.00133) and dysgeusia (p = 0.000539) scores at day 10 of the follow-up period. Relative treatment effect (RTE) plots for CT value (based on the E and ORF genes) and anosmia and dysgeusia scores, against a follow-up period, for the two groups was evaluated with Wald statistics followed by ANOVA (Fig. 2). Fig. 2 demonstrates relative treatment effect on Y axis and days of follow up on the X axis. It is plotted for the four study parameters – E gene, ORF gene, anosmia and Dysgeusia.

## 4 Discussion

The present study was carried out to explore the plant-based phytochemical's preliminary evaluation in the clinical management of COVID-19. Almost all drug options tried in COVID-19 have produced unclear efficacy evidence except the steroids. Initial safety assessment in a single and multiple ascending dose pattern showed good tolerability with all the doses and frequencies of EO blend nebulization administered. The participants did not complain of hypersensitivity, rash, pain, breathing difficulty, or discomfort. One patient in the level six safety cohort mentioned a mild burning sensation during EO nebulization, which resolved independently without requiring any rescue medication or further investigation. This finding shows that EO blend nebulization appears to be safe for patient use in the nebulized form. Horvath et al. demonstrated that the volatile nature of these EOs makes them more suitably delivered to the entire respiratory tract in nebulized form with NS dilution. Additional anti-inflammatory action may provide symptomatic relief to the patient taking it via the inhalation route. Plant-based EOs are regularly used in tea leaves, room fresheners, traditional medicine, and food ingredients, especially in the South Indian diet. The health benefits of EOs are mentioned in Ayurveda, an ancient Indian medical system, for its antiasthmatic, mucolytic, anti-inflammatory, decongestant, and antimicrobial actions [8]. COVID-19 study participants coming to the hospital's emergency unit were initially subjected to a 6-min walk test as per hospital emergency medicine COVID protocol. COVID-19 patients not showing oxygen desaturation on the 6-min walk test were labeled as having mild COVID-19 disease and advised home-based care. Participants agreeing to give informed consent out of this pool were considered for Phase II of the study.

The present study saw the evolution of the new COVID-19 symptoms, and general respiratory symptoms were significantly lower with EO blend nebulization in asymptomatic RT-PCR-positive patients at baseline. The ingredients of the present study's EO blend, such as menthol and eucalyptus oil, are proven to be soothing for respiratory discomfort [9]. Peppermint oil, one of the constituents in the present EO blend, is known to help in digestive disorders, cold, and cough. At the same time, another ingredient is tea tree oil, which is known to benefit patients suffering from influenza, cold, and bronchitis. However, the safety of these oils in pregnancy remains inadequately studied to date [10,11].

Symptomatic mild COVID-19 disease participants who received EO nebulization could not show a statistically significant benefit in COVID-19 symptom evolution compared to the placebo. This may be explained by the absence of therapeutic benefits or the reconsideration required for the dose. On the other hand, asymptomatic individuals developing fewer symptoms while on EO nebulization demonstrate a potential prophylactic role. Valussi et al. recently exercised caution on the controlled use of herbal medicines in COVID-19, reiterating symptomatic benefits and the absence of therapeutic confirmation [9]. The potential of phytochemicals in COVID-19 has been suggested by in vitro studies and computer-aided molecular docking studies [12,13]. Taking it ahead in the initial phase of clinical trial design is the most challenging task, as the dose, frequency per day, and latency in the onset of action may differ indication-wise [14]. The plant-based phytochemicals in the study medication (i.e., EO blend) may have drug interactions that can be antagonistic or synergistic. Simultaneous evaluation of each phytochemical ingredient separately and in the presence of others, in varying proportions, may increase the translational value of potential phytochemicals [15].

The study further explored the effect of EO nebulization on viremia clearance and general respiratory symptom remission. The study observed a statistically insignificant difference between two groups with respect to E and ORF gene CT scoring on follow-up days 5 and 10. In the present study, the in vitro antiviral action of EO ingredients was not translated in vivo with a specific EO blend formula. The study findings suggest symptomatic benefits and the absence of antiviral activity with EO blend nebulization at specific doses studied here. The composition of the EO blend may need reconsideration for concentration-specific ingredients in clinical evaluation [16]. Anosmia and dysgeusia, assessed using a visual analog scale, showed statistically significant improvements with EO blend nebulization compared to the placebo. The results of the present study confirm symptomatic benefits as mentioned in the literature and Ayurvedic practice. Thus, it can be considered an add-on drug or individually for anosmia, dysgeusia, and the prevention of symptoms of COVID-19. The anosmia and dysgeusia of other etiologies might also benefit from EO nebulization.

### 4.1 Limitations

Moderate to severe COVID-19 patients were not studied for therapeutic exploration of EO blend nebulization. No formal Phase II dose calculation was done, as dose-limiting toxicities were not reached in the Phase I doses studied.

## 5 Conclusion

EO blend nebulization prevented the evolution of COVID-19 symptoms and provided symptomatic benefit in anosmia and dysgeusia but not viraemia clearance with doses studied here.

## Author contributions

All research done by the authors. ANP and RR designed the study, drafted the initial manuscript, RM, AV, obtained the patient consent form, collected data, and filed the patient case. ANP prepared a herbarium and approved the final manuscript. ALVKR, not a part of the investigator team, designed, prepared and standardized a condensed essential oil blend for study purpose. RR, RM, AV, AK, NH randomized patients, analyzed data and revised the manuscript. ALVKR was involved in the preparation of final phytopharmaceutical, essential oil blend and placebo. RM, VS, AB, RK, KG, AG, NG confirmed the diagnosis of COVID-19 patients and revised the manuscript. AA and SS took care of logistic support between the Department of Pharmacology and Department of Internal medicine. APS and ANP supervised the various stages of study, revised and approved the final manuscript. All authors have confirmed the submitted manuscript and are responding to study.

## Funding

None.

## Declaration of competing interest

None.

## Figures and Tables

**figs1:**