# Supplementary material for: Essential oil nebulization in mild COVID-19(EONCO): Early phase exploratory clinical trial
Source: J Ayurveda Integr Med. 2022 Jul 6;13(3):100626. doi: 10.1016/j.jaim.2022.100626 (PMC9257088; doi:10.1016/j.jaim.2022.100626)
Supplement: Multimedia component 1 [file mmc1.docx]

**Supplementary file Table 1: Components of the Essential oil (EO) blend and their properties**

| **S.No** | ***E O[Total volume 125 ml]*** | **Plant species** | **Properties** | **Composition** | **Common beneficial effects proposed in Ayurvedic practice** |  |
| --- | --- | --- | --- | --- | --- | --- |
| **1** | ***Lemon Oil***  ***(10 ml)*** | Citrus limon | Density: 0.849-0.858  (849-858 mg/ml)  RI: 1.470 – 1.476 | Terpinenes (94%), Limonene  Sesquiterpenes,  aldehydes (citrals, about 3.4-3.6%)  15 geranyl acetate | - Adsorbent (has the capacity to bind pollutants) - Contains limonoids (reduces cholesterol lipid lowering, reduces cancer) - Alkaloids - Has citric effect( vitamin C) - Dilating property - Opens bronchioles |  |
| **2** | ***Oregano Oil***  ***(10 ml)*** | Origanum vulgare | Density: 0.977  (977 mg/ml) | Carvacrol(63.97%),  p-cymene(12.63%), linalool(3.67%),  α-terpineol(999992.54%),  terpinen-4-ol(2.24%) | - Should contain carvacrol at least 90% to ascertain the purity of the oil - Mucolytic property - Decongestant - Expectorant property |  |
| **3** | ***Tea Tree Oil***  ***(5 ml)*** | Melaleuca alternifolia | Density: 0.885-0.906  (885-906 mg/ml)  RI: 1.475 – 1.482 | Terpinen-4-ol(48.0%),  y-terpinene(28.0%),1,8-cineole(15.0%),  α-terpinene(13.0%), α-terpineol(8.0%),   p-cymene(8.0%), sabinene(3.5%) | - Procured from New Zealand and Australia are the best - Dries mucus - Has the property to allow other medicines to sink in - Opens bronchioles - Has penetrating property - Anti-fungal - Anti-bacterial - Anti-viral - Has absorbent ( soaks up and dries up) quality |  |
| **4** | ***Java Citronella Oil***  ***(20 ml)*** | Cymbopogon winterianus Jowitt | Density: 0.875-0.893  (875-893 mg/ml)  RI: 1.466 – 1.474 | Limonene (6.07%), linalool (1.04%), citronellal (50.93%), citronellol (6.06%), geraniol (16.47%), citronellyl acetate (3.89%), geranyl acetate (4.94%), alpha-selinene (1.40%), gamma-cadinene (1.06%), elemol (1.15%). and beta-caryophyllene oxide (1.15%) | - Chemical composition of java citronella oil and its properties. - Insect repellent - Bacteria repellent - Microbe repellent |  |
| **5** | ***Turmeric***  ***(10 ml)*** | Curcuma longa | Density: 0.930 – 0.945 @ 25^o^C  (930-945 mg/ml)  RI: 1.502-1.513 | Sesquiterpenes, like a-phellandrene , b-Caryophyllene, b-farnesene .;ar-termerone; b-esquiphellandrene ,ar-curcumene ,d-3-carene ,specific for  a spices. | - Anti-bacterial - Kills microbes - Anti-inflammatory - Does not allow fungus to grow - Analgesic - Anti-pyretic - Controls temperature |  |
| **6** | ***Peppermint***  ***(10 ml)*** | Mentha piperita | Density: 0.856- 0.946  (856-946 mg/ml)  RI: 1.384-1.530 | High menthol content (44.39%), along with menthone (15.36%), and other essential oils such as menthyl acetate (4.78%), 1,8-cineole (5.3%), limonene (1.87%), menthofuran (10.27%), and β-caryophyllene (1.7%). | - South Africa – amazon wild peppermint oil best - Mucolytic - Cooling effect - Decongestant |  |
| **7** | ***Lavender***  ***(5 ml)*** | Lavandula | Density: 0.875- 0.888  (875-888 mg/ml)  RI: 1.458-1.468 | linalool (30.6 %),  linalyl acetate (14.2 %), geraniol (5.3 %),  β-caryophyllene (4.7 %),  lavandulyl acetate (4.4 %). | - Best if procured from Kashmir. - Insect repellent - Dilator - Anti-inflammatory - Analgesic - (having bath by adding 2-3 drops in bucket full of water has a very soothing effect) |  |
| **8** | ***Ginger***  ***(5 ml)*** | Zingiber officinalis | Density: 0.885- 0.900 @ 25^o^C (885-900 mg/ml)  RI: 1.485- 1.500 @ 20^o^C | The essential oil was characterized by high percentage of sesquiterpenes (66.66%), monoterpenes (17.28%) and aliphatic compounds (13.58%). The predominant sesquiterpene was zingiberene (46.71%) followed by valencene (7.61%), β-funebrene (3.09%) and selina-4(14),7(11)-diene (1.03%). The major monoterpenes were characterized as citronellyl n-butyrate (19.34%), β-phellandrene (3.70%) | - Should contain 98% gingerol - Anti-inflammatory - Expectorant - Carmative (reduces flatulence) - Mucolytic - Very potent and should be used in small quantities - Anti-spasmodic |  |
| **9** | ***Frankincense***  ***(10 ml)*** | Boswellia carteri Birdw | Density: 0.865 @ 25^o^C (865 mg/ml)  RI: 1.468 | [α-pinene](https://www.sciencedirect.com/topics/agricultural-and-biological-sciences/alpha-pinene) (2.0–64.7%); α-thujene (0.3–52.4%); [β-pinene](https://www.sciencedirect.com/topics/agricultural-and-biological-sciences/beta-pinene) (0.3–13.1%); [myrcene](https://www.sciencedirect.com/topics/agricultural-and-biological-sciences/myrcene) (1.1–22.4%); sabinene(0.57.0%); [limonene](https://www.sciencedirect.com/topics/agricultural-and-biological-sciences/limonene) (1.3–20.4%);  *p*-cymene (2.7–16.9%) and  β-caryophyllene (0.1–10.5%) | - Disinfectant - Anti-microbial - Calms mind - Produces serotonin - Decongestant - Tussive (prevents or relieves cough) - Purifies the atmosphere. |  |
| **10** | ***Eucalyptus***  ***(5 ml)*** | Eucalyptus  Myrtaceae | Density: 0.906- 0.927 (906-927 mg/ml)  RI: 1.458- 1.470 | 1,8-cineol (49.07 to 83.59%)  α-pinene (1.27 to 26.35%) | - Should be prepared only from tender leaves of the tree - Expectorant - Decongestant - Broncho dilator - Antimicrobial - Insect repellent and also acts as absorbent |  |
| **11** | ***Wheat Germ***  ***(5 ml)*** | Triticum Aestivum L | Density: 0.91 – o.93 @ 20^o^C (910-930 mg/ml) | Hexanal (15.97%), 2-methyl-2-butene (10.43%), 2,4- heptadienal (8.53%), and limonene (6.83%).  The oil was rich in unsaturated fatty acid (83.45%), especially in linoleic acid (64.82%), and there were 75.49%  β-sitosterol (64.64%). | - Contains six types of tocopherols (property of vitamin E) - Smoothening effect for the bronchioles |  |
| **12** | ***Basil***  ***(5 ml)*** | Ocimum basilicum L. | Density: 0.880-0.911 (880-911 mg/ml)  RI: 1.470-1.490 | Methylcinnamate (70.1%),  linalool (17.5%), β-elemene (2.6%) and  camphor (1.52%).anisole (1.5%) | - Immune booster - T cell activity increases - Immune modulator - Anti-microbial - Anti-inflammatory |  |
| **13** | ***Cedar Wood***  ***(5 ml)*** | Juniperus virginiana | Density: 0.940- 1.000 @ 20^o^C  0.925 @ 25^o^C  (940-1000 mg/ml)  RI: 1.493- 1.513 | Terpinen-4-ol (36%), sabinene (19.2%),   γ-terpinene (10.4%), α-terpinene (5.5%) and   myrcene (5.5%) thujopsene (= widdrene) (47.1%),  α-cedrol (10.7%), widdrol (8.5%) and cuparene (4.0%) | - Best place to procure is Kashmir. - Produces warmthness - Has the property to reduce effects of type B u-v rays - Anti-inflammatory - Has the property to protect the keratin layer which in turn protects the outer skin and makes it waterproof. - Protects the outer layer of the bronchiole - Anti-microbial - Has high conductivity |  |
| **14** | ***Holy basil***  ***(5 ml)*** | Ocimum tenuiflorum | Density: 0.965- 0.985 (965-985 mg/ml)  RI: 1.490- 1.520 | Eugenol (1.94–60.20 %), methyl eugenol (0.87–82.98 %), β-caryophyllene (4.13–44.60 %),  β-elemene (0.76–32.41 %). | - Antiviral activity |  |
| **15** | ***Cinnamon***  ***(5 ml)*** | Cinnamomum verum | Density: 1.030- 1.050 (1030-1050 mg/ml)  RI: 1.529- 1.540 | Cinnamaldehyde and eugenol,   coumarin, camphor,  linalool | - To be used in very small quantities - Has all the above properties - Sri Lanka – best place to procure this oil. |  |
| **16** | ***Sage oil***  ***(5 ml)*** | Salvia officinalis | Density: 0.886- 0.929 (886-929 mg/ml)  RI: 1.458 – 1.473 | Linalyl acetate, Linalol,  beta-caryophyllene,  alpha-terpineol, Geraniol | - Antimicrobial action - Antidepressant action - Analgesic action |  |
| **17** | ***Clove oil***  ***(5 ml)*** | Syzygium aromaticum | Density: 1.034- 1.048 (1034- 1048 mg/ml)  RI: 1.525- 1.535 | Eugenol (80-85%)  Caryophyllene  Alpha Lumulene  Caryophyllene oxide  Acetyl Eugenol | - Antimicrobial action - Anti cough , anti asthmatic action - analgesic action |  |

**Supplementary table 2: Vaccination for COVID-19 received by the study participants in each arm.**

|  | **Arm A (AT)** | **Arm B (ST)** | **Arm C (AP)** | **Arm D (SP)** | **Total** | **p value** |
| --- | --- | --- | --- | --- | --- | --- |
| **COVID-19 Vaccine** [Frequency (Percentage)] | | | | | | |
| Vaccinated | 7 (43.8) | 18 (36) | 3 (37.5) | 13 (52) | 41 (41.4) | 0.599^ |
| Not vaccinated | 9 (56.3) | 32 (64) | 5 (62.5) | 12 (48) | 58 (58.6) |  |
| **Number of Doses** [Frequency (Percentage)] | | | | | | |
| Both doses | 2 (12.5) | 3 (6) | 1 (12.5) | 4 (16) | 10 (10.1) | 0.742^ |
| Only One dose | 5 (31.3) | 15 (30) | 2 (25) | 9 (36) | 31 (31.3) |  |
| Not vaccinated | 9 (56.3) | 32 (64) | 5 (62.5) | 12 (48) | 58 (58.6) |  |
| **Time interval from vaccination to testing positive for COVID-19** (in days) [Median (Interquartile range)] | | | | | | |
| Time interval | 0 (0 – 26.5) | 0 (0 – 13) | 0 (0 – 9.5) | 6 (0 – 21) | 0 (0 – 16.5) | 0.700^@^ |

**^** Fisher Exact test used. ^@^Kruskal-Wallis test used. Significance level is 0.05


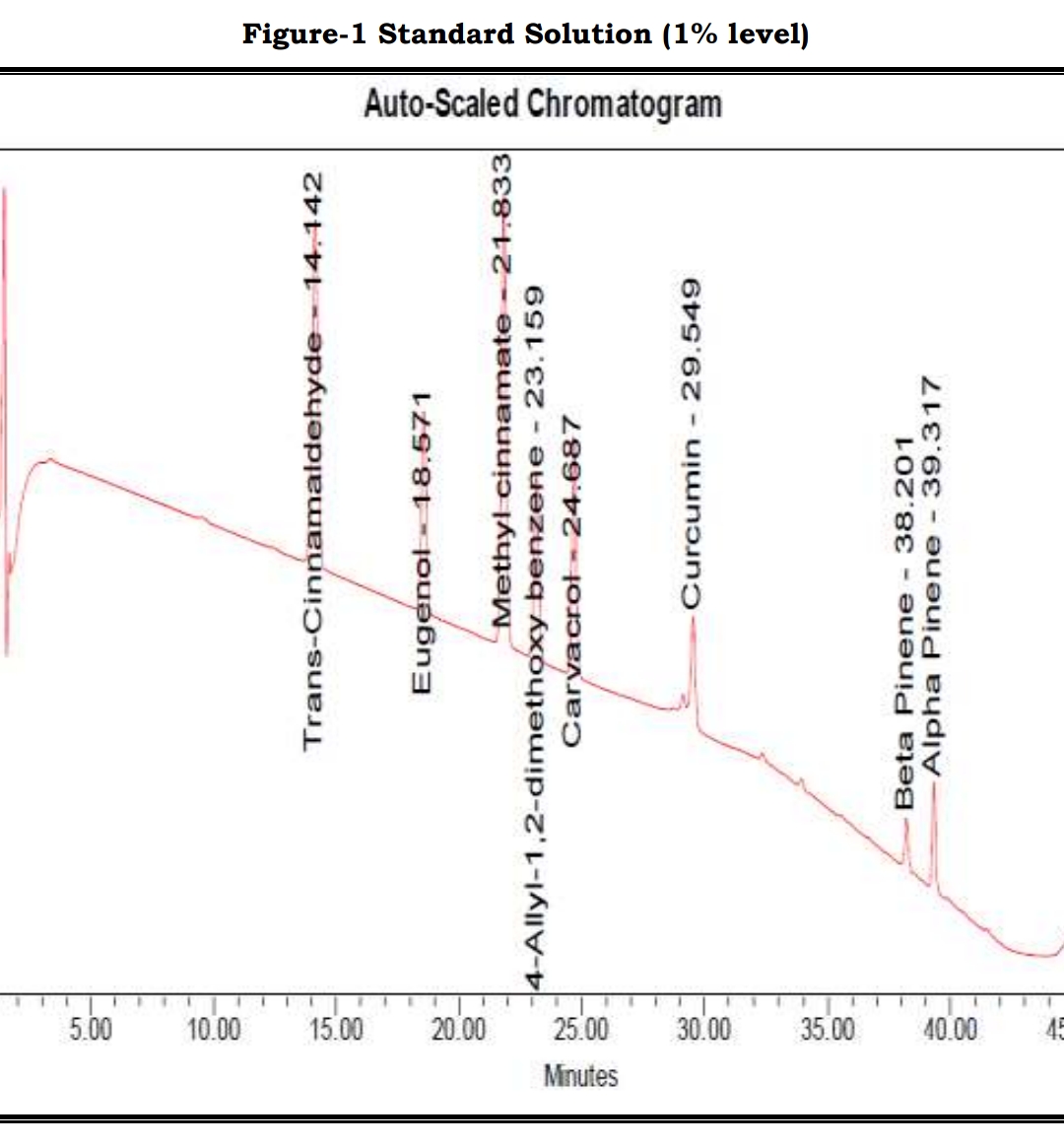


**Supplementary file Figure 1:** Typical Chromatogram showing peaks of some of the Phytochemical constituents, present in the mixture at 220nm wavelength.
